# Accessibility audit of the Midwest Chapter of the Medical Library Association

**DOI:** 10.5195/jmla.2025.2092

**Published:** 2025-10-23

**Authors:** JJ Pionke, Carol Shannon, Matthew Regan, Gerald Natal, Jessica D. Gilbert Redman, Jessica Decaro, Anna Biszaha, Julia Carol Stumpff, Jennifer Feldman, Sara Westall

**Affiliations:** 1 pionke@umich.edu, Instructor, Syracuse University, Syracuse, NY; 2 shannon@umich.edu, Informationist, Taubman Health Sciences Library, University of Michigan, Ann Arbor, MI; 3 matthew-regan@uiowa.edu, University of Iowa, IA; 4 gerald.natal@utoledo.edu, University of Toledo, Toledo, OH; 5 jdgr.library@gmail.com, University of North Dakota, ND; 6 jessica.decaro@case.edu, Case Western Reserve University; 7 anna.biszaha@osumc.edu, Ohio State University, Cinncinati, OH; 8 jstumpff@iu.edu, Indiana University, Indianapolis, IN; 9 feldmaj@ccf.org, Cleveland Clinic, Cleveland, OH; 10 sara.westall@und.edu, University of North Dakota, ND

**Keywords:** Disability, accessibility, Midwest Chapter of the Medical Library Association, presidential project, task force, project management, policy

## Abstract

**Background:**

In 2023, JJ Pionke became President of the Midwest Chapter of the Medical Library Association (MWCMLA). He determined that for his presidential year, he would form a task force to determine the accessibility levels of the chapter and remediate accessibility issues as appropriate.

**Case Presentation:**

To accomplish the accessibility audit of the MWCMLA, Pionke formed an Accessibility Task Force that was time limited to one year. Task force meetings were held once a month to keep people accountable and to share out progress and requests for assistance. The task force was broken up into four teams: annual meeting, policy, social media, and website. Task force members could be on more than one team. The goals of each team were generally the same: what are other organizations doing, what do we have already if anything, and develop best practices/policy/etc. as needed.

**Conclusions:**

The teams fulfilled their mandate by creating best practices/guidelines/policies documents. Some accessibility remediation was needed for the chapter website. The task force's findings and materials were shared out among the MWCMLA as well as passed on to the presidents of the other chapters, many of whom had expressed interest in the results.

## BACKGROUND

While librarianship has long been interested in accessibility for people with disabilities, even before the Americans with Disabilities Act (ADA) was signed into law in 1990, the profession has recently taken a much keener interest in improving accessibility of our spaces and services for both patrons and employees. Libraries have leaned heavily on ADA for accessibility of physical spaces. There are several pieces of legislation, in addition to ADA, that cover digital accessibility including but not limited to Section 508 of the Rehabilitation Act of 1973. Likewise the Web Content Accessibility Guidelines (WCAG) is the major website accessibility guidelines followed worldwide. As interest in accessibility has grown within the profession, keeping in mind that accessibility is beyond ADA compliance, there has been growing concern over how accessible library organizations are both internally in terms of documents like meeting minutes, and externally such as organization websites and conferences. This case study focuses on an accessibility audit that was conducted by the Midwest Chapter of the Medical Library Association (MWCMLA) of its policies regarding accessibility in various domains such as the annual meeting, social media, and general accessibility policy as well as the accessibility of the MWCMLA's website.

## CASE PRESENTATION

In 2023, JJ Pionke began his term as President of the MWCMLA. He determined that for his presidential year he wanted to create a one year time limited task force to conduct an accessibility audit of the organization. Pionke's research expertise is in disability and accessibility for both library patrons and employees so leading the accessibility audit was a natural fit for him. He sent out a recruitment email to the MWCMLA email listserv. The entire group of people interested in doing the work met and then broke up into teams to address the four separate areas of need: annual meeting, policy, social media, and website. Members of the task force could be on any team they wished and could be on more than one team. Pionke largely acted as the project manager for the entire project by scheduling and running monthly meetings, acting as an expert consultant, and kept the entire task force time and project aware. The teams met individually on their own schedules and typically sent at least one representative to a monthly task force meeting for checking in, getting advice and assistance, brainstorming, etc. At the end of the year-long task force, the team brought together their individual outputs such as social media best practices as well as task force outputs like an executive summary that was distributed to the entire MWCMLA and also disseminated to the other Chapter Presidents. Each team had slightly different scopes depending on what their broader accessibility focus was. Below is a breakdown of what each team focused on and the outputs that they created.

### Annual Meeting Team

The annual meeting team (Carol Shannon, Gerald Natal, and Ximena Chrisagis) began by surveying the websites of a variety of library organizations. While there were many resources listed, only one set of older guidelines from 2016 was discovered. The team next turned their focus to university websites, specifically those of the University Library and the Office of Diversity, Equity, and Inclusion, both at the University of Michigan, and the Accessible Meeting and Event Checklist of Cornell University. All three sites were comprehensive, clear, and included information on in-person and virtual meetings, including information for the conference (before and during the meeting) and for presenters and participants.

The team created two documents, one for in-person and one for virtual conferences, to make it easier for the conference planners to see what it was they needed to do for accessibility. The documents also contain links to useful resources for conference planners and participants. Some guidelines will need to be reconsidered, as the 2025 conference for the MWCMLA will be in a hybrid format (virtual but with the possibility of an institution hosting a “watch party” to recreate some of the activities that we miss from in-person conferences like catered receptions for networking).

All sections of the documents have been assigned to specific conference planning subcommittees, so that everyone involved should understand which subcommittee is responsible for which part of planning. The MWCMLA Mixers, a quarterly “lunch and learn” webinar, hosted an informal discussion on accessibility and the conference for members to learn more about the new accessibility policies.

### Policy Team

The policy team (Julia Stumpff, Sara Westall, and Jennifer Feldman) evaluated all MWCMLA websites looking for accessibility policies. They then reached out to all chapters to see if they had any accessibility policies not found on chapter websites. No chapter had an accessibility policy.

The committee then conducted a literature search on accessibility policies and drafted a chapter policy based on findings from the literature search [1–5]. The draft policy was presented to the accessibility task force for feedback and edits. Next, it was taken to the MWCMLA Board for suggestions and edits.

Once approved, the accessibility policy was posted on the chapter website and will be reviewed annually by the Communication and IDEA committees.

### Social Media Team

The social media team (JJ Pionke, Jessica D. Gilbert Redman, and Jessica DeCaro) completed an exploratory evaluation of social media policies in other organizations. Given the wide range of social media options available today and the ever-changing social media landscape, the group decided to create a best practices guide, providing details on specifics to make social media posts as accessible as possible for a wide variety of platforms (e.g., using alt text for images wherever the platform allows). In addition to the generalized advice, the team also included links to specific accessibility pages to common social media platforms, both those that the MWCMLA currently uses and ones that may be useful in the future.

Given the unpredictable nature of social media changes, it is expected that this resource will be a living document and updated regularly to include appropriate information from current social media platforms and processes.

### Website Team

The website team (Matt Regan, Anna Biszaha, and Jessica D. Gilbert Redman) completed a digital accessibility audit of all public-facing pages of the MWCMLA website with the goal of bringing the site into compliance with WCAG 2.2 AA standards. To complete this audit, the team used the WebAIM WAVE Web Accessibility Evaluation Tool and then logged any accessibility issues in a tracking spreadsheet. Over 125 issues were reported by WebAIM's WAVE, including errors, alerts, structural elements, and webpage features. Each reported issue was evaluated, a determination was made whether a change was truly needed, and solutions were researched. Access to edit the website was granted to each team member so that when a solution was identified and could be implemented, the team was able to immediately fix errors.

Many errors were determined to be caused by how Wild Apricot, which is the CMS used to publish the website, formats content. First, it was discovered that the native header construction and navigation menu caused many accessibility issues that repeated on every page of the website. Due to this, a new page template was created using custom HTML and CSS that could be applied to every web page. This new template included a modified login button, site search form, and adjustments to the image used for the logo. Second, Wild Apricot uses “gadgets” to organize and format content on the website, but uses tables to do so, which was flagged as an accessibility issue. It became necessary to delete gadgets where possible and reorganize content using custom HTML. Lastly, Wild Apricot organizes pages into the navigation menu as they are created through page settings. That navigation menu automatically generates title attributes for each link which matches the name of the webpage, which was flagged as an accessibility issue. There are no methods in the system to remove those attributes, so it was necessary to contact the Wild Apricot development team, which led to the creation of a JavaScript string that corrected this issue site-wide. Other site-wide accessibility issues, including contrast errors for navigation links, were corrected using the CSS customization options.

Many additional errors were fixed on a page-by-page basis. Because much of the content in the site was imported as raw HTML from the previous version, there were prolific heading structure issues to correct. Broken links were identified and corrected or removed, and link text was streamlined to improve consistency in navigation. During this process, content was updated, duplicate information consolidated, and consistent formatting applied to related pages to improve usability.

There are still a number of accessibility issues that have not, or can not, be resolved during the scope of this project. The most widespread issue is the presence of PDFs and Word documents linked throughout the website. It was determined that evaluating these documents for accessibility was outside the scope of the website team, and the large number made it impractical for the web accessibility task force to correct at this time. The team recommends that future committee chairs receive training to create accessible PDFs for any future documents to be uploaded to the website. One of the more impactful issues is that the top menu cannot be fully utilized through keyboard navigation, which will be a definite barrier to anyone accessing the site through a screen reader. This issue cannot be addressed without the intervention of the Wild Apricot development team, as the navigation menu is automatically generated by the software. There are also a few form label issues, such as the login form, which are automatically generated and unable to be edited.

Overall, the accessibility team feels it has greatly improved the accessibility and usability of the website, within the limitations of the website software. However, it has become apparent that future efforts to improve accessibility should include advocating for Wild Apricot to address the inherent accessibility issues of their software, or to consider other alternatives. This process has led to a consensus among team members that a permanent website team may be warranted and that periodic reviews of the site will be necessary to maintain compliance.

### Actionable Ideas

- Scope: Be very clear and specific about what the scope of the project is. Is the team doing an assessment only or are they empowered to create policy/best practices documents/fix digital objects, etc.
- Project Management: Be robust with project management and time management skills. For example, be clear on how long the committee/task force/working group is going to be together. Set regular deadlines and standing meetings for checking in. Have a project manager who schedules and runs all of the meetings to keep everyone on track, on task, and on target.
- Many Hands Make Light Work: Recruit a sufficiently sized group and then break the group up into teams based on where their interests are. Have at least one person from each team be a representative to monthly check-in meetings.
- Create Tangible Outputs Both Internally and Externally to the Organization: In the case of this project, each team produced an output whether it was a policy document or a best practices list or making the website more accessible. However, working within academia means that certain outputs are more favored than others for tenure and/or promotion. To that end, there were additional outputs that could be directly pointed to in curriculum vitaes/annual reports/promotion and/or tenure papers. These outputs were an executive summary for dissemination to the MWCMLA and to the Presidents of other Chapters, a poster suitable for conferences that anyone on the task force could use, and a case report article for an academic journal of the entire project.

## CONCLUSION

The accessibility audit of the MWCMLA was successful in large part because there was a team of people that self-selected into the project due to their interest in either learning accessibility skills or applying those skills or both. The four teams each had a scope and support throughout the year-long process as well as a scaffolded structure which included deadlines and regular meetings. The audit drew a lot of interest from both within the MWCMLA and other Chapters because accessibility is a topic that is becoming more discussed within librarianship and there is a need within the profession to be more accessible. A clear scope, strong project and time management, and a group of willing professionals who wanted to learn more and contribute to bettering the organization, is what made the accessibility audit of the MWCMLA a success.
